# Supplementary material for: Comparing Large Language Model AI and Human-Generated Coaching Messages for Behavioral Weight Loss
Source: J Technol Behav Sci. 2025 Feb 24;10(4):749–60. doi: 10.1007/s41347-025-00491-5 (PMC12816013; doi:10.1007/s41347-025-00491-5)
Supplement: Supplementary file 1 — Supplementary file1 (DOCX 26 KB) [file 41347_2025_491_MOESM1_ESM.docx]

**Supplementary Appendix**

**Table 1.** Human coach written messages.

| **Message Number** | **Human-written Messages** |
| --- | --- |
| 1 | You pulled together a nice loss on the scale this past week! I know you’ve been struggling with some regain lately, but your hard work weighing in each day, tracking your food and limiting your calories really paid off this week. Nice job tracking your calories more days this week than last week! What would help you to increase your food tracking from 5/7 days all the way up to 7/7 days this next week? Consider setting yourself a reminder or an alarm on your phone, or focus on items on those other 2 days that are very easy to track like pre-packaged meals. |
| 2 | Wow, you knocked it out of the park again this week with weight loss! One of the biggest contributors to your success is likely that you’re tracking your calories every single day. This is such a critical behavior for achieving and maintaining weight loss that it’s truly hard to overestimate its importance. Give yourself a pat on the back for a job well done these past few weeks, and I’m confident you’ll be able to keep it up this upcoming week. |
| 3 | I see you had a small gain this past week, which is not at all uncommon after a big loss like the one you had the week before. It might just be an off week, given that you’ve been so successful with weight loss and have generally been losing lately. But it does also seem like your activity was a little lower this week than last time, which could be part of what’s going on. What can you do to increase those activity minutes next time? Perhaps consider scheduling out your activity in advance using a calendar or planner, or if you’re getting bored with the activity you’re doing, maybe try out an activity you haven’t tried in a while… whether that’s swimming, biking, hiking in nature or jumping jacks! |
| 4 | Seems like this was a tougher week as far as the scale goes, which I know can be disappointing after the success you had last week. I 100% believe in your ability to still succeed in this program — let’s think about what you haven’t tried yet that could help get you back on track. The biggest thing that jumps out to me is your calorie totals, which were above your calorie goal on a few days this past week. Is there anything higher-calorie in your diet right now that you’d be willing to consume less of or remove entirely if you’re not feeling especially attached to it? Common examples might include milk/sugar in coffee or tea, late-night salty snacks (crunchy vegetables can substitute for these in a pinch) and snacks with added sugar like granola bars (whole fruit can be a good substitute here!). |
| 5 | You maintained your weight this week, after some fluctuations this past month. Based on your terrific overall weight loss success, I’m quite confident you can push past this mini-plateau and keep losing weight; sometimes it just takes a few weeks! In the meantime, what are some additional areas you can focus on? It seems like physical activity was a bit lower this time than last time, and we know from research that high levels of activity are strongly predictive of maintaining your weight loss. So, I’d consider pushing your activity back up towards that 250 minute mark this upcoming week — consider scheduling this time out in advance using a calendar or planner, and treating it like you would any other work, family or social commitment. |

**Table 2.** The input prompts and AI-generated messages in Phase 1.

| **Message Number** | **Input Prompt** | **AI-generated Messages** |
| --- | --- | --- |
| 1 | You are a weight loss coach in a 52-week behavioral weight loss program. The participant’s goal in the program is to lose 10% of body weight. This is week 41, and below is a summary of the participant’s data and the data pattern in the past three weeks:  1. Overall, the participant gained 1.10% of their body weight. The person’s daily calorie goal ranges from 1350 to 1650 calories. 2. In the past week, the person lost 0.4 lbs of body weight. The person tracked their weight for 7 days. The person tracked their calories 5 out of 7 days. Among the days the person tracked, they were in their calorie range for 0 days, above their calorie range for 4 days, and under their calorie range for 1 day. Their physical activity goal was to have 250 minutes of moderate to vigorous activity, and they did 29 minutes of moderate to vigorous activity. 3. Two weeks ago, the person gained 1 lbs of body weight. The person tracked their calories 4 out of 7 days. Among the days the person tracked, they were in their calorie range for 0 days, above their calorie range for 4 days, and under their calorie range for 0 days. Their physical activity goal was to have 250 minutes of moderate to vigorous activity, and they did 25 minutes of moderate to vigorous activity. 4. Three weeks ago, the person gained 2 lbs of body weight. The person tracked their calories 2 out of 7 days. Among the days the person tracked, they were in their calorie range for 0 days, above their calorie range for 2 days, and under their calorie range for 0 day. Their physical activity goal was to have 250 minutes of moderate to vigorous activity, and they did 63 minutes of moderate to vigorous activity.  Write no more than 5 sentences of a message directed to the participant that summarizes the data and the data pattern you see in the past three weeks, praise and validate the part that is going well, point out things that are not going well, and provides helpful strategies to address the part that is not going well and put them in the form of implementation intentions. You will first comment on weight data and patterns. You will then comment on behavior data and patterns by only picking and commenting on one of the four behaviors domains and none of the others, including physical activity, food tracking, self-weighing, or calories, that the participant needs to work on the most. | I notice that you have gained 1.0% of your body weight overall, which means we need to refocus on our weight loss goals. However, it's great to see that you lost 0.4 lbs last week! Over the past three weeks, I noticed a pattern of not consistently tracking your calories. Last week, you only tracked your calories 5 out of 7 days, and two weeks ago, you only tracked your calories 4 out of 7 days. To address this issue, try setting implementation intentions to track your calories every day, even if it means logging them in advance. Plan to use reminders on your phone or set a specific time each day to log your meals. Consistent tracking is key to meeting your weight loss goals. |
| 2 | You are a weight loss coach in a 52-week behavioral weight loss program. The participant’s goal in the program is to lose 10% of body weight. This is week 8, and below is a summary of the participant’s data and the data pattern in the past three weeks:  1. Overall, the participant lost 6.90% of their body weight. The person’s daily calorie goal ranges from 1200 to 1500 calories. 2. In the past week, the person lost 1.2 lbs of body weight. The person tracked their weight for 7 days. The person tracked their calories 7 out of 7 days. Among the days the person tracked, they were in their calorie range for 3 days, above their calorie range for 4 days, and under their calorie range for 0 day. Their physical activity goal was to have 175 minutes of moderate to vigorous activity, and they did 312 minutes of moderate to vigorous activity. 3. Two weeks ago, the person lost 2 lbs of body weight. The person tracked their calories 7 out of 7 days. Among the days the person tracked, they were in their calorie range for 2 days, above their calorie range for 4 days, and under their calorie range for 1 day. Their physical activity goal was to have 150 minutes of moderate to vigorous activity, and they did 422 minutes of moderate to vigorous activity. 4. Three weeks ago, the person lost 0.6 lbs of body weight. The person tracked their calories 7 out of 7 days. Among the days the person tracked, they were in their calorie range for 4 days, above their calorie range for 3 days, and under their calorie range for 0 days. Their physical activity goal was to have 120 minutes of moderate to vigorous activity, and they did 297 minutes of moderate to vigorous activity.  Write no more than 5 sentences of a message directed to the participant that summarizes the data and the data pattern you see in the past three weeks, praise the part that is going well, point out things that are not going well, and provides helpful strategies to address the part that is not going well and put them in the form of implementation intentions. You will first comment on weight data and pattern and then comment on behavior data and pattern. For the behavioral part of the message, only pick and comment on one of the four behaviors domains (i.e., physical activity, food tracking, self-weighing, or calories) that the participant needs to work on the most. | Congratulations on losing 6.9% of your body weight! Your weight loss pattern has been positive, with losses of 1.2 lbs, 2 lbs, and 0.6 lbs over the past three weeks. However, you are consistently exceeding your daily calorie range. To improve in this area, try setting specific and measurable goals, such as eating within your calorie range for 5 out of 7 days each week. Plan ahead and make healthy food choices, such as packing a nutritious lunch, snacking on fruits and vegetables, and cooking at home instead of eating out. These steps will help you stay on track and reach your weight loss goals. |
| 3 | You are a weight loss coach in a 52-week behavioral weight loss program. The participant’s goal in the program is to lose 10% of body weight. This is week 27, and below is a summary of the participant’s data and the data pattern in the past three weeks:  1. Overall, the participant lost 14.2% of their body weight. The person’s daily calorie goal ranges from 1200 to 1500 calories. 2. In the past week, the person gained 0.8 lbs of body weight. The person tracked their weight for 7 days. The person tracked their calories 7 out of 7 days. Among the days the person tracked, they were in their calorie range for 0 days, above their calorie range for 7 days, and under their calorie range for 0 days. Their physical activity goal was to have 250 minutes of moderate to vigorous activity, and they did 133 minutes of moderate to vigorous activity. 3. Two weeks ago, the person lost 3.8 lbs of body weight. The person tracked their calories 7 out of 7 days. Among the days the person tracked, they were in their calorie range for 1 day, above their calorie range for 6 days, and under their calorie range for 0 day. Their physical activity goal was to have 250 minutes of moderate to vigorous activity, and they did 141 minutes of moderate to vigorous activity. 4. Three weeks ago, the person gained 0.4 lbs of body weight. The person tracked their calories 7 out of 7 days. Among the days the person tracked, they were in their calorie range for 1 day, above their calorie range for 6 days, and under their calorie range for 0 day. Their physical activity goal was to have 250 minutes of moderate to vigorous activity, and they did 317 minutes of moderate to vigorous activity.  Write no more than 5 sentences of a message directed to the participant that summarizes the data and the data pattern you see in the past three weeks, praise the part that is going well, point out things that are not going well, and provides helpful strategies to address the part that is not going well and put them in the form of implementation intentions. You will first comment on weight data and pattern and then comment on behavior data and pattern. For the behavioral part of the message, pick one of the four behavior domains (physical activity, food tracking, self-weighing, or calories) and comment on the one the participant needs to work on the most. | Congratulations on losing 14.2% of your body weight so far! However, in the past three weeks, there seems to be a pattern of not staying within your calorie range. Last week, you were above your calorie range for 7 days, which could be the reason for your 0.8 lb weight gain. In two weeks ago, you were also above your calorie range for 6 days and gained 0.4 lbs three weeks ago. To address this issue, try setting implementation intentions to prioritize staying within your daily calorie range. Make a plan to have healthy snack options readily available and to prepare meals in advance to ensure you are sticking to your calorie goals. |
| 4 | You are a weight loss coach in a 52-week behavioral weight loss program. The participant’s goal in the program is to lose 10% of body weight. This is week 39, and below is a summary of the participant’s data and the data pattern in the past three weeks:  1. Overall, the participant gained 0.7% of their body weight. The person’s daily calorie goal ranges from 1350 to 1650 calories. 2. In the past week, the person gained 2 lbs of body weight. The person tracked their weight for 2 days. The person tracked their calories 2 out of 7 days. Among the days the person tracked, they were in their calorie range for 0 days, above their calorie range for 2 days, and under their calorie range for 0 day. Their physical activity goal was to have 250 minutes of moderate to vigorous activity, and they did 63 minutes of moderate to vigorous activity. 3. Two weeks ago, the person lost 1.2 lbs of body weight. The person tracked their calories 4 out of 7 days. Among the days the person tracked, they were in their calorie range for 1 day, above their calorie range for 2 days, and under their calorie range for 0 days. Their physical activity goal was to have 790 minutes of moderate to vigorous activity, and they did 47 minutes of moderate to vigorous activity. 4. Three weeks ago, the person gained 1 lbs of body weight. The person tracked their calories 4 out of 7 days. Among the days the person tracked, they were in their calorie range for 1 day, above their calorie range for 3 days, and under their calorie range for 0 days. Their physical activity goal was to have 776 minutes of moderate to vigorous activity, and they did 36 minutes of moderate to vigorous activity.  Write no more than 5 sentences of a message directed to the participant that summarizes the data and the data pattern you see in the past three weeks, praise the part that is going well, point out things that are not going well, and provides helpful strategies to address the part that is not going well and put them in the form of implementation intentions. You will first comment on weight data and pattern and then comment on behavior data and pattern. For the behavioral part of the message, only pick and comment on one of the four behaviors domains, including physical activity, food tracking, self-weighing, or calories, that the participant needs to work on the most. | Great job on tracking your weight for the past three weeks! Unfortunately, the data shows that you have gained weight in the past week and have had an overall gain of 0.7% of your body weight. To improve your weight loss journey, it's important to focus on tracking your food intake accurately. Implement the strategy of tracking your calories every day, aiming to be within your calorie range of 1350 to 1650 calories. This will help you to stay on track and reach your goal of losing 10% of your body weight. |
| 5 | You are a weight loss coach in a 52-week behavioral weight loss program. The participant’s goal in the program is to lose 10% of body weight. This is week 37, and below is a summary of the participant’s data and the data pattern in the past three weeks:  1. Overall, the participant lost 10.20% of their body weight. The person’s daily calorie goal ranges from 1200 to 1500 calories. 2. In the past week, the person's weight remained unchanged. The person tracked their weight for 6 days. The person tracked their calories 6 out of 7 days. Among the days the person tracked, they were in their calorie range for 3 days, above their calorie range for 2 days, and under their calorie range for 1 day. Their physical activity goal was to have 250 minutes of moderate to vigorous activity, and they did 148 minutes of moderate to vigorous activity. 3. Two weeks ago, the person lost 1.9 lbs of body weight. The person tracked their calories 3 out of 7 days. Among the days the person tracked, they were in their calorie range for 2 days, above their calorie range for 1 day, and under their calorie range for 0 days. Their physical activity goal was to have 250 minutes of moderate to vigorous activity, and they did 303 minutes of moderate to vigorous activity. 4. Three weeks ago, the person gained 2.1 lbs of body weight. The person tracked their calories 5 out of 7 days. Among the days the person tracked, they were in their calorie range for 0 days, above their calorie range for 1 day, and under their calorie range for 4 days. Their physical activity goal was to have 250 minutes of moderate to vigorous activity, and they did 67 minutes of moderate to vigorous activity.  Write no more than 5 sentences of a message directed to the participant that summarizes the data and the data pattern you see in the past three weeks, praise the part that is going well, point out things that are not going well, and provides helpful strategies to address the part that is not going well and put them in the form of implementation intentions. You will first comment on weight data and pattern and then comment on behavior data and pattern. For the behavioral part of the message, only pick and comment on one of the four behaviors domains, including physical activity, food tracking, self-weighing, or calories, that the participant needs to work on the most. | Great job maintaining your weight this week! It's great to see that you have been keeping up with self-weighing, tracking your calories 6 out of 7 days, and staying within your calorie range for 3 of those days. However, your physical activity level is still below your goal of 250 minutes of moderate to vigorous activity. To improve this, let's set an implementation intention to schedule physical activity for at least 30 minutes each day after work or before breakfast. This will help you reach your goal of 250 minutes of moderate to vigorous activity and support your weight loss journey. |

**Table 3.** The input prompts and AI-generated messages in Phase 2.

| **Message Number** | **Input Prompt** | **AI-generated Message** |
| --- | --- | --- |
| 1 | You are a weight loss coach in a 52-week behavioral weight loss program. The participant’s goal in the program is to lose 10% of body weight. This is week 41, and below is a summary of the participant’s data and the data pattern in the past three weeks:  1. Overall, the participant gained 1.10% of their body weight. The person’s daily calorie goal ranges from 1350 to 1650 calories. 2. In the past week, the person lost 0.4 lbs of body weight. The person tracked their weight for 7 days. The person tracked their calories 5 out of 7 days. Among the days the person tracked, they were in their calorie range for 0 days, above their calorie range for 4 days, and under their calorie range for 1 day. Their physical activity goal was to have 250 minutes of moderate to vigorous activity, and they did 29 minutes of moderate to vigorous activity. 3. Two weeks ago, the person gained 1 lbs of body weight. The person tracked their calories 4 out of 7 days. Among the days the person tracked, they were in their calorie range for 0 days, above their calorie range for 4 days, and under their calorie range for 0 days. Their physical activity goal was to have 250 minutes of moderate to vigorous activity, and they did 25 minutes of moderate to vigorous activity. 4. Three weeks ago, the person gained 2 lbs of body weight. The person tracked their calories 2 out of 7 days. Among the days the person tracked, they were in their calorie range for 0 days, above their calorie range for 2 days, and under their calorie range for 0 day. Their physical activity goal was to have 250 minutes of moderate to vigorous activity, and they did 63 minutes of moderate to vigorous activity.  Write a very encouraging and empathetic message with touches of humor to the participant to provide feedback based on the criteria below: the message must be 5 sentences or less in a single paragraph; the message should use a second-person pronoun; the message should summarize the data and pattern in the past three weeks; you should praise and validate the part that is going well, if any, with excitement and touches of humor, empathetically pointing out things that are not going well, and provide helpful strategies to address the part that is not going well and putting them in the form of implementation intentions by using very encouraging and light-hearted language; the message should first comment on weight data and patterns. The message will then summarize the data and comment on only one of the four behavior domains and none of the others: physical activity, food tracking, self-weighing, or calories the participant needs to work on the most. For example, if you comment on food tracking, don’t comment on physical activity, self-weighing, or calories. | Congratulations on losing weight this week! That's fantastic progress! Looking at the past three weeks, we noticed that you've had some ups and downs with your weight. While it's natural to have some fluctuations, let's work on getting back on track together. It seems like food tracking has been a bit of a challenge for you, but don't worry, we have some ideas to make it easier. Try prepping your meals ahead of time or using a food journal to keep yourself accountable. You got this! |
| 2 | You are a weight loss coach in a 52-week behavioral weight loss program. The participant’s goal in the program is to lose 10% of body weight. This is week 8, and below is a summary of the participant’s data and the data pattern in the past three weeks:  1. Overall, the participant lost 6.90% of their body weight. The person’s daily calorie goal ranges from 1200 to 1500 calories. 2. In the past week, the person lost 1.2 lbs of body weight. The person tracked their weight for 7 days. The person tracked their calories 7 out of 7 days. Among the days the person tracked, they were in their calorie range for 3 days, above their calorie range for 4 days, and under their calorie range for 0 day. Their physical activity goal was to have 175 minutes of moderate to vigorous activity, and they did 312 minutes of moderate to vigorous activity. 3. Two weeks ago, the person lost 2 lbs of body weight. The person tracked their calories 7 out of 7 days. Among the days the person tracked, they were in their calorie range for 2 days, above their calorie range for 4 days, and under their calorie range for 1 day. Their physical activity goal was to have 150 minutes of moderate to vigorous activity, and they did 422 minutes of moderate to vigorous activity. 4. Three weeks ago, the person lost 0.6 lbs of body weight. The person tracked their calories 7 out of 7 days. Among the days the person tracked, they were in their calorie range for 4 days, above their calorie range for 3 days, and under their calorie range for 0 days. Their physical activity goal was to have 120 minutes of moderate to vigorous activity, and they did 297 minutes of moderate to vigorous activity.  Write a very encouraging and empathetic message with touches of humor to the participant to provide feedback based on the criteria below: the message must be 5 sentences or less in a single paragraph; the message should use a second-person pronoun; the message should summarize the data and pattern in the past three weeks; you should praise and validate the part that is going well, if any, with excitement and touches of humor, empathetically pointing out things that are not going well, and provide helpful strategies to address the part that is not going well and putting them in the form of implementation intentions by using very encouraging and light-hearted language; the message should first comment on weight data and patterns. The message will then summarize the data and comment on only one of the four behavior domains and none of the others: physical activity, food tracking, self-weighing, or calories the participant needs to work on the most. For example, if you comment on food tracking, don’t comment on physical activity, self-weighing, or calories. | Great job on losing 6.90% of your body weight! Keep up the fantastic work! It's awesome that you lost 1.2 lbs this week! It's not always easy to stay within your calorie range, but you tracked your calories every day, and that's a great start. Let's try to hit your calorie range every day this week! When you feel tempted to go above your calorie range, take a deep breath and remind yourself of your goal to lose 10% of your body weight. You're doing amazing, and I believe in you! Let's make it happen! Don't forget to track your food, stay within your calorie range, and keep up the good work! |
| 3 | You are a weight loss coach in a 52-week behavioral weight loss program. The participant’s goal in the program is to lose 10% of body weight. This is week 27, and below is a summary of the participant’s data and the data pattern in the past three weeks:  1. Overall, the participant lost 14.2% of their body weight. The person’s daily calorie goal ranges from 1200 to 1500 calories. 2. In the past week, the person gained 0.8 lbs of body weight. The person tracked their weight for 7 days. The person tracked their calories 7 out of 7 days. Among the days the person tracked, they were in their calorie range for 0 days, above their calorie range for 7 days, and under their calorie range for 0 days. Their physical activity goal was to have 250 minutes of moderate to vigorous activity, and they did 133 minutes of moderate to vigorous activity. 3. Two weeks ago, the person lost 3.8 lbs of body weight. The person tracked their calories 7 out of 7 days. Among the days the person tracked, they were in their calorie range for 1 day, above their calorie range for 6 days, and under their calorie range for 0 day. Their physical activity goal was to have 250 minutes of moderate to vigorous activity, and they did 141 minutes of moderate to vigorous activity. 4. Three weeks ago, the person gained 0.4 lbs of body weight. The person tracked their calories 7 out of 7 days. Among the days the person tracked, they were in their calorie range for 1 day, above their calorie range for 6 days, and under their calorie range for 0 day. Their physical activity goal was to have 250 minutes of moderate to vigorous activity, and they did 317 minutes of moderate to vigorous activity.  Write a very encouraging and empathetic message with touches of humor to the participant to provide feedback based on the criteria below: the message must be 5 sentences or less in a single paragraph; the message should use a second-person pronoun; the message should summarize the data and pattern in the past three weeks; you should praise and validate the part that is going well, if any, with excitement and touches of humor, empathetically pointing out things that are not going well, and provide helpful strategies to address the part that is not going well and putting them in the form of implementation intentions by using very encouraging and light-hearted language; the message should first comment on weight data and patterns. The message will then summarize the data and comment on only one of the four behavior domains and none of the others: physical activity, food tracking, self-weighing, or calories the participant needs to work on the most. For example, if you comment on food tracking, don’t comment on physical activity, self-weighing, or calories. | You're doing great! You've lost an impressive 14.2% of your body weight so far, which is amazing! However, over the past three weeks, there have been some bumps in the road. Although you lost weight two weeks ago, you gained a little last week, and three weeks ago, your weight remained almost the same. To help you get back on track, let's focus on your physical activity. You've been doing well with tracking your calories, but you've fallen a little short on your physical activity goals. To get back on track, let's set an implementation intention to increase your moderate to vigorous physical activity by 10 minutes every other day. Remember, every small step counts! |
| 4 | You are a weight loss coach in a 52-week behavioral weight loss program. The participant’s goal in the program is to lose 10% of body weight. This is week 39, and below is a summary of the participant’s data and the data pattern in the past three weeks:  1. Overall, the participant gained 0.7% of their body weight. The person’s daily calorie goal ranges from 1350 to 1650 calories. 2. In the past week, the person gained 2 lbs of body weight. The person tracked their weight for 2 days. The person tracked their calories 2 out of 7 days. Among the days the person tracked, they were in their calorie range for 0 days, above their calorie range for 2 days, and under their calorie range for 0 day. Their physical activity goal was to have 250 minutes of moderate to vigorous activity, and they did 63 minutes of moderate to vigorous activity. 3. Two weeks ago, the person lost 1.2 lbs of body weight. The person tracked their calories 4 out of 7 days. Among the days the person tracked, they were in their calorie range for 1 day, above their calorie range for 2 days, and under their calorie range for 0 days. Their physical activity goal was to have 790 minutes of moderate to vigorous activity, and they did 47 minutes of moderate to vigorous activity. 4. Three weeks ago, the person gained 1 lbs of body weight. The person tracked their calories 4 out of 7 days. Among the days the person tracked, they were in their calorie range for 1 day, above their calorie range for 3 days, and under their calorie range for 0 days. Their physical activity goal was to have 776 minutes of moderate to vigorous activity, and they did 36 minutes of moderate to vigorous activity.  Write a very encouraging and empathetic message with touches of humor to the participant to provide feedback based on the criteria below: the message must be 5 sentences or less in a single paragraph; the message should use a second-person pronoun; the message should summarize the data and pattern in the past three weeks; you should praise and validate the part that is going well, if any, with excitement and touches of humor, empathetically pointing out things that are not going well, and provide helpful strategies to address the part that is not going well and putting them in the form of implementation intentions by using very encouraging and light-hearted language; the message should first comment on weight data and patterns. The message will then summarize the data and comment on only one of the four behavior domains and none of the others: physical activity, food tracking, self-weighing, or calories the participant needs to work on the most. For example, if you comment on food tracking, don’t comment on physical activity, self-weighing, or calories. | Hey there! You're doing great with the program, and I'm proud of you for sticking with it this long! We've all had some bumps in the road, and your recent weight gain is just that - a bump. Let's get back on track together! I know it can be tough to track calories every day, but it's essential to stay in your range. Let's try setting an implementation intention: "I will track my calories every day for the next week, no matter what." Remember, it's not about perfection, it's about progress. Let's keep pushing forward! |
| 5 | You are a weight loss coach in a 52-week behavioral weight loss program. The participant’s goal in the program is to lose 10% of body weight. This is week 37, and below is a summary of the participant’s data and the data pattern in the past three weeks: 1. Overall, the participant lost 10.20% of their body weight. The person’s daily calorie goal ranges from 1200 to 1500 calories. 2. In the past week, the person's weight remained unchanged. The person tracked their weight for 6 days. The person tracked their calories 6 out of 7 days. Among the days the person tracked, they were in their calorie range for 3 days, above their calorie range for 2 days, and under their calorie range for 1 day. Their physical activity goal was to have 250 minutes of moderate to vigorous activity, and they did 148 minutes of moderate to vigorous activity. 3. Two weeks ago, the person lost 1.9 lbs of body weight. The person tracked their calories 3 out of 7 days. Among the days the person tracked, they were in their calorie range for 2 days, above their calorie range for 1 day, and under their calorie range for 0 days. Their physical activity goal was to have 250 minutes of moderate to vigorous activity, and they did 303 minutes of moderate to vigorous activity. 4. Three weeks ago, the person gained 2.1 lbs of body weight. The person tracked their calories 5 out of 7 days. Among the days the person tracked, they were in their calorie range for 0 days, above their calorie range for 1 day, and under their calorie range for 4 days. Their physical activity goal was to have 250 minutes of moderate to vigorous activity, and they did 67 minutes of moderate to vigorous activity.  Write a very encouraging and empathetic message with touches of humor to the participant to provide feedback based on the criteria below: the message must be 5 sentences or less in a single paragraph; the message should use a second-person pronoun; the message should summarize the data and pattern in the past three weeks; you should praise and validate the part that is going well, if any, with excitement and touches of humor, empathetically pointing out things that are not going well, and provide helpful strategies to address the part that is not going well and putting them in the form of implementation intentions by using very encouraging and light-hearted language; the message should first comment on weight data and patterns. The message will then summarize the data and comment on only one of the four behavior domains and none of the others: physical activity, food tracking, self-weighing, or calories the participant needs to work on the most. For example, if you comment on food tracking, don’t comment on physical activity, self-weighing, or calories. | Congratulations on your incredible achievement of losing 10.20% of your body weight! You've come a long way, and that's something to be proud of. Although your weight has remained unchanged this week, it's fantastic that you tracked your weight and calories for six days! Let's focus on increasing your physical activity to reach your goal of 250 minutes of moderate to vigorous activity per week. You've done an amazing job in the past, and I know you can do it again! Let's set an implementation intention for the week: "I will walk for 30 minutes after dinner every day this week." Keep up the excellent work, and I'm looking forward to seeing your progress next week! |

**Table 4.** Themes and representative extracts.

| **Themes** | **AI-generated messages feel more formulaic and impersonal, with less encouragement of autonomy.** | **Participants desire messages that offer validation and motivational support, complemented by specific and personalized recommendations for behavioral change.** | **Participants seek messages to consider the full context of their data trends to offer more targeted behavior suggestions.** |
| --- | --- | --- | --- |
| **Representative Extract #1** | *They come across as being more formula-like and scripted and pick up on the deficits. (3004)* | *It's a little overly positive for me for a week that looks like I didn't hit many goals. I would like more blunt honesty. (1013)* | *I think (for me), the problem is the assumption component and lack of context. What if this person was on vacation - that would be a great week. (1014)* |
| **Representative Extract #2** | *[The human coach message] seems more understanding of the struggles, but recognizes and encourages the positive behaviors. (1029)* | *I have had weeks like this where i am feeling pretty bummed about my progress and getting a message like this is so encouraging and motivating for me to want to continue the program even if i am not seeing the loss that I would like to see. (1023)* | *At Week 39, with an overall gain, I'd suspect that unless I had something medically or emotionally going on with me causing this gain, I'd simply lose interest in the program. Just telling me to track at this point without supportive suggestions wouldn't be motivating. I would probably not change my habits as a result of this message if I hadn't already. (3062)* |
| **Representative Extract #3** | *This message sounds curious ("It might be this...it could be related to this...") which, to me, feels engaging and respectful of my competence and self-awareness. (1008)* | *It seems more effective to focus on the positive first and then provide the specific ways to improve. (2067)* | *There is very little encouragement and you're assuming the person isn't focused on their weight loss goals... just because people aren't meeting their calorie goals doesn't mean they aren't trying or don't feel bad about it. (3009)* |
| **Representative Extract #4** | *[AI messages are] data heavy and formal in tone. (3021)* | *It's somewhat generic, and the same concepts are readily available elsewhere. (1038)* | *I like the reference to the big picture, and calling this a "mini-plateau." And it did ask what my ideas were, before suggesting the physical activity idea, with the further ideas about a planner/ calendar. (1031)* |
| **Representative Extract #5** | *[AI messages] just didn't have the feeling of "I'm your coach and we're going to figure this out together." (3039)* | *It is positive and sets an intention, but it needs to be more specific. i.e. "I will track daily and aim to consume x calories daily. I will do that by cutting out sugar in my coffee and eating while watching Netflix." (1040)* | *[The message] would be a good reminder of how important tracking is but may not get at the reason why tracking wasn't completed. For example, an illness, family emergency, etc. (3020)* |
